# Use of Information and Communication Technology (ICT) Devices Among the Oldest-Old: Loneliness, Anomie, and Autonomy

**DOI:** 10.1093/geroni/igz050

**Published:** 2020-01-01

**Authors:** Anna Schlomann, Alexander Seifert, Susanne Zank, Christiane Woopen, Christian Rietz

**Affiliations:** 1 Department of Special Education and Rehabilitation Science, Faculty of Human Sciences, University of Cologne, Germany; 2 University Research Priority Program “Dynamics of Healthy Aging”, Switzerland; 3 Center of Competence for Gerontology, University of Zurich, Switzerland; 4 Cologne Center for Ethics, Rights, Economics, and Social Sciences of Health (CERES), Germany; 5 Research Unit Ethics, Faculty of Medicine and University Hospital Cologne, University of Cologne, Germany; 6 Mixed Methods Research, Faculty of Educational and Social Sciences, Heidelberg University of Education, Germany

**Keywords:** Digitization, Germany, Internet, Subjective well-being, Technology use

## Abstract

**Background and Objectives:**

A good person–environment-fit has positive effects on well-being in old age. As digital technologies are an integral part of older adults’ environments, we predicted that the use of information and communication technologies (ICT) is associated with subjective well-being among the oldest-old. Specifically, we compared different user groups of ICT devices (nonusers, users of nonweb-connected ICT, users of web-connected ICT) and analyzed the relations among ICT use and three domains of subjective well-being (loneliness, anomie, autonomy).

**Research Design and Methods:**

We performed a quantitative data analysis using data from the first representative state-wide survey study in North-Rhine Westphalia, Germany on quality of life and well-being of the oldest-old (*n* = 1,698; age range: 80–103; 9% long-term care). Multiple regression analyses were applied.

**Results:**

The findings revealed that 25.9% of all individuals aged 80 years and older reported using web-connected ICT, in contrast to 38.5% who do not use ICT at all. Individuals who used web-connected ICT reported lower levels of loneliness and anomie, and higher levels of autonomy. These differences remain significant when controlling for indicators of social inclusion and individual characteristics.

**Discussion and Implications:**

This study investigated an underexplored group in terms of ICT use, shedding light on the relationship between ICT use and subjective well-being. The oldest-old generally use ICT in their everyday life but an age-related digital divide still exists. To avoid negative consequences of nonuse digital infrastructures and technology training for older adults need to be established.

**Translational Significance**

The use of information and communication technologies (ICT) is positively related to well-being among the oldest-old even when considering indicators of social inclusion and individual characteristics. Highlighting such positive effects may increase ICT use in advanced age. To realize the benefits of ICT use among the oldest-old technology training and digital infrastructures are necessary.

## Background and Objectives

Digital technologies have become an integral part of modern societies and the environments of older individuals (Wahl & Gerstorf, 2018). One important trend has been the spread of information and communication technologies (ICT; Castells, 2010).

ICT include devices and applications that provide access to information and enable electronic communications, like sending text messages or engaging in video chats. Mobile phones, smartphones, computers, and laptops are typical ICT devices. The Internet (e.g., the web) is another ICT and plays a special role because it is not a stand-alone device but a network of countless systems and devices. Web-connected ICT devices (e.g., smartphones, tablets) are defined by their access to the Internet (web connection), whereas nonweb-connected ICT (e.g., old mobile phones) do not access the Internet.

ICT use in general may enable older individuals to live independently for a longer time and may have positive effects on health and social isolation (Czaja, Boot, Charness, Rogers, & Sharit, 2018; Schulz et al., 2015). Web-connected ICT provides new communication capabilities. Its use facilitates higher levels of interaction and broader access to digital information and services than nonweb-connected ICT. Research has shown that web-connected ICT has substantially changed everyday life by enabling new forms of social participation and interaction, and enhanced access to information (e.g., Antonucci, Ajrouch, & Manalel, 2017; Castellacci & Tveito, 2018; Kim, Lee, Christensen, & Merighi, 2017). Against this background, the distinction between the two types of ICT (i.e., web-connected and nonweb-connected) is important when analyzing the role of ICT in older adults’ everyday lives. The diffusion of ICT in society is an ongoing process (Hill, Betts, & Gardner, 2015). Still, many older adults lag in the ownership of web-connected ICT and Internet use compared with the general population (Mitzner et al., 2019; Pew Research Center, 2018). There are further differences between the young-old and the oldest-old. Using European data, König, Seifert, and Doh (2018) showed that less than 10% of those aged 80 and older accessed the Internet, whereas 48% of Europeans aged 65–69 did.

In this way, the ongoing digitization of everyday life and differences in technology adoption can lead to new inequalities in society (Francis, Ball, Kadylak, & Cotten, 2019). The oldest-old individuals who do not use web-connected ICT devices risk being socially excluded from a society that is characterized by a diffusion of digital innovations (Cotten, 2017; Olsson, Samuelsson, & Viscovi, 2017). Research has demonstrated positive associations between web-connected ICT use and older adults’ well-being (e.g., Cotten et al., 2014, Szabo, Allen, Stephens, & Alpass, 2019; Quintana, Cervantes, Sáez, & Isasi, 2018). However, there is limited knowledge for the group of the oldest-old. There are initial findings that demonstrate higher levels of well-being for users of digital technology in the sample case of digital games in very old age (Allaire et al., 2013), but representative data on these topics is still scarce. As there are unique challenges in researching this group, the oldest-old are often underrepresented in large-scale survey studies (Davies et al., 2010). Available public statistics often overestimate the percentages of older ICT users because the statistics are mostly based on samples of community-dwelling older adults and do not consider older adults who live in institutional care settings (Cotten, 2017).

Our study addresses these gaps in the research and analyzes the relationship between ICT use (web-connected and nonweb-connected) and subjective well-being in very old age. Based on a representative sample of the oldest-old cohorts (80+) and with reference to environmental gerontology (Wahl & Gerstorf, 2018; Wahl, Iwarsson, & Oswald, 2012) we focus on three domains of subjective well-being: loneliness, anomie, and autonomy. These aspects were selected because they represent a relevant and broad spectrum of subjective well-being in old age and their relation with technology use among the oldest-old population have been theoretically discussed (Czaja et al., 2018; Schulz et al., 2015; Sims, Reed, & Carr, 2017).

### Use of ICT and Loneliness

Loneliness is defined as the subjective experience of involuntary social isolation or the feeling of being alone (Cotten, Anderson, & McCullough, 2013). ICT devices are designed for social interaction over long distances; and social networking applications, sending text messages, and engaging in video chats provide opportunities for social contact. In this way, modern technology can enable new forms of social interaction especially for older adults (Antonucci et al., 2017). The empirical evidence on the relationship between loneliness and ICT use in old age is mixed. Some studies identified reduced loneliness (e.g., Cotten et al., 2013; Lelkes, 2013; Szabo et al., 2019). A systematic review demonstrated that the use of computers and the Internet in randomized controlled trial designs was not significantly related to lower levels of loneliness (Chen & Schulz, 2016). These mixed results might be related to the question of whether real-life social interactions are replaced by interactions via ICT, which might lead to higher experiences of loneliness. However, a review on social relations and technology concludes that technology is more likely to expand traditional forms of social interactions than to replace them (Antonucci, et al., 2017).

### Use of ICT and Anomie

Anomie describes an individual’s disorientation or aimlessness, which is characterized by feelings of nontransparency of personal and social interrelations (Gümüs, Gömleksiz, Glöckner-Rist, & Balke, 2014). In today’s modern societies, an increasing amount of information, and services can only be accessed online using web-connected ICT. The Internet offers the opportunity to stay up to date on the latest news, make purchases, or carry out financial transactions. People without access to the Internet are systematically excluded and not using web-connected ICT means not being able to access certain information or use certain services. This, in turn, may affect people’s daily lives and evoke feelings of exclusion from the (digital) society (Hill et al., 2015; Seifert, Hofer, & Rössel, 2018).

ICT use can create social participation in old age, for example, by volunteering with community organizations (Kim et al., 2017). By accessing online information about the society they live in, individuals may also have a stronger sense of belonging to the society and their community, which may reduce feelings of anomie (Castellacci & Tveito, 2018).

### Use of ICT and Autonomy

Autonomy refers to individuals’ capacity to decide how to act that makes them the “authors of most of their actions, and [..] thus accountable for most of what they do” (Buss & Westlund, 2018). This includes a presence of choices in terms of opportunities (e.g., “I live my life according to my own ideas”); however, more opportunities do not necessarily mean greater autonomy. The breadth of ICT use was associated with fewer functional limitations in a sample of individuals aged 80+ (Sims et al., 2017), and fewer functional limitations might be positively related to their subjective assessment of autonomy. The use of web-connected ICT for instrumental purposes such as online shopping, banking, or seeking health information may also increase perceptions of autonomy in older adults (Elliot, Mooney, Douthit, & Lynch, 2014). Some older adults use ICT because they hope that these technologies can maintain their autonomy in later life (Hernández-Encuentra, Pousada, & Gómez-Zúñiga, 2009; Seifert & Schelling, 2018).

### Conceptual Framework

We analyze these relationships between ICT use and selected domains of subjective well-being within the conceptual framework of environmental gerontology. A central aim of environmental gerontology is to examine the contribution of person–environment exchange processes on different outcomes such as well-being, autonomy, identity, and health (Wahl et al., 2012). Environments are not restricted to physical or “built” contexts but also include social environments and technologies (Wahl & Gerstorf, 2018). Fundamental changes in the composition of the context have occurred within the technological realm. In this way, person–technology interactions are an important research field. Aging individuals have to adapt continuously to the environment they live in that has become characterized by increasing digitization, and to their own changing capacities (Wahl & Gerstorf, 2018).

Although contexts can enrich or restrain functionality and development throughout the lifespan, new technologies can be an enrichment of the environment and at the same time represent new requirements for the (aging) individual. When the fit between older persons and their environment is beneficial, they enjoy opportunities to build and maintain their intrinsic capacity and functional ability (Lawton, 1982).

We expect a more beneficial person–environment fit when a person uses modern technologies because the person has successfully adapted to the changing technological context. According to the assumptions of environmental gerontology, this good person–technology fit would lead to positive effects on well-being. Nonuse might increase the probability that individuals perceive their digital environment as exclusionary rather than stimulating. However, empirical evidence on these processes is scarce. There are promising results on the effects of technology on health-related outcomes (Schulz et al., 2015) but there is not very much research that examines the impact of the technological context on well-being in old age (Wahl & Gerstorf, 2018). In a review article, Forsman, Nordmyr, Matosevic, Wahlbeck, and McDaid (2017) identified favorable effects of computer and Internet training on older adults’ mental well-being but they also report a lack of high-quality studies. Against this background, one aim of the emerging field of environmental aging and technology research is to study “[…] whether and how technology serves as a window to the world during times when functional limitations become more frequent and severe and thereby contribute to maintaining well-being” (Wahl & Gerstorf, 2018, p. 16).

### Current Study

We assume that the relationships between ICT use and subjective well-being are different for web-connected ICT (e.g., smartphones or tablets) and nonweb-connected ICT (e.g., mobile phones or PCs without access to the Internet) because Internet access allows for broader access to information and interactive connectivity. From an environmental gerontology perspective, the person–environment fit is less beneficial when a person does not use web-connected ICT because the individual is less adapted to the (digital) environment. Thus, not using web-connected ICT in a digital society might lead to feelings of anomie, loneliness, and decreased autonomy because individuals are more likely to perceive a gap between their own life and society. The relationship between ICT use and loneliness has been inconclusive in previous research and needs to be studied in more detail. Although some studies have demonstrated that Internet use was related to lower levels of loneliness in older adults, others had nonsignificant results. To the best of our knowledge, the relationship between ICT use and anomie has not yet been researched among the oldest-old. Using ICT for instrumental purposes can contribute to higher levels of independence and foster autonomy. However, we could not identify any studies that have analyzed these relationships for different kinds of ICT (i.e., web-connected and nonweb-connected) nor with a sample of oldest-old individuals. As digital technologies are now an integral part of older adults’ environments, we hypothesized that individuals (80+) who use web-connected ICT report

(1) lower levels of loneliness,
(2) lower levels of anomie, and
(3) higher levels of autonomy

than those who use nonweb-connected ICT or who do not use any ICT devices.

## Research Design and Method

### Data and Study Sample

This paper reports the results of a secondary analysis based on data from the NRW80+ project. The NRW80+ project is the first representative state-wide survey study on quality of life and subjective well-being of the oldest-old (80+) undertaken in the federal state of North Rhine-Westphalia (NRW) in Germany (Wagner et al., 2018). NRW is the federal state with the largest population in Germany and has about 18 million inhabitants, of whom more than 1 million are age 80 or older. NRW is often described as a small Germany: with its large industry, structurally weak regions, and medium-sized wealth, the federal state reflects the whole of Germany. The population density of NRW is comparatively high with an average of 525.1 inhabitants per square kilometer (Destatis, 2019). About 90% of households have access to the Internet (IT.NRW, 2019). For the NRW80+ survey study, multistage sampling was applied with a random sample of individuals aged 80 years and older in private homes and institutional settings. The study design of NRW80+ builds on a preceding feasibility study in which all survey material was piloted (Wagner et al., 2018). The preliminary study also served to determine the maximum reasonable interview duration. As a result, an average interview duration of 90 min was envisaged. If necessary, scales were adapted to the life context of the oldest-old and shortened in order to achieve an appropriate interview duration.

In total, 1,863 computer-assisted personal interviews were realized, including 165 interviews with proxy informants. The interviews were conducted in German; for the purpose of this paper, all reported terminology and questions were translated from German into English by the authors. For the analyses, only the personal interviews with the target individuals are considered, resulting in a sample of 1,698 interviews. Of those, 1,539 are community-dwelling older adults and 156 live in institutional care settings. The mean duration of the interviews was 86 min. The study was approved by the Ethics Committee of the Medical Faculty of the University of Cologne (No. 17-169), and all of the participants gave informed consent.

### Measures

#### Dependent variables

Loneliness was measured using a single-item question (based on the Center for Epidemiologic Studies Depression scale by Andresen, Malmgren, Carter, & Patrick, 1994) “How often did you feel lonely during the last week?” with a four-point scale (“never or almost never,” “sometimes,” “mostly,” and “always or almost always”), and higher values indicated higher levels of loneliness. The estimated prevalence of loneliness in this study is comparable to other German aging survey studies (Luhmann & Bücker, 2019).

Anomie was measured using a three-item scale (“Do you have the feeling that you are coping less and less with today’s social way of life?,” “Do you have the feeling that your own values are becoming less and less compatible with the values of today’s society?,” and “Do you have the feeling that today’s society is changing so quickly that you no longer know how to orient yourself in society?”). The items were developed with reference to the scale proposed by Gümüs et al. (2014). All three items were measured using four-point Likert scales (“does not apply at all” to “fully applies”), and the scale was calculated by adding the respective points of the three items and dividing it by the number of items. Higher values indicate higher feelings of anomie. The internal consistency was found to be moderate in the current sample, with a Cronbach’s alpha of 0.67. This value is comparable to the Cronbach’s alpha reported for the original anomie scale by Gümüs et al. (2014).

Autonomy was measured (based on the perceived autonomy in old age scale by Schwarzer, 2008) using one question, “Do you lead your life according to your own ideas?” with a four-point Likert scale (“does not apply at all” to “fully applies”). Higher values indicate a higher level of autonomy. Although multi-item measurements are generally preferred, one-item measures are an economic method in survey research.

#### Independent variables

The study included some questions on technology use that were used to define three ICT user groups. All of the participants were asked whether they use (yes/no) a PC/laptop, mobile phone, smartphone, tablet, an activity tracker, and the Internet. Three groups were then developed for the analyses:

1. *No ICT use (* ***no ICT***): individuals who do not use any of the aforementioned ICT.
2. *Use of nonweb-connected ICT (* ***nonweb ICT***): individuals who use a mobile phone or a PC/laptop but do not access the Internet, and do not use a smartphone, tablet, or fitness tracker.
3. *Use of web-connected ICT (* ***web ICT***): individuals who access the Internet and use at least one of the following: a smartphone, a tablet, or a fitness tracker.

#### Control variables

Further control variables were considered in the analyses. We included indicators of social inclusion because these indicators may be related to the experience of loneliness, anomie, and autonomy. Individuals who score higher on indicators of social inclusion might perceive lower levels of loneliness and anomie, and higher levels of autonomy because they are less likely to experience gaps between their own life and society. The level of social inclusion may also be related to ICT use. However, it is unknown if ICT interactions in very old age replace or complement real-life social interactions (Antonucci et al., 2017). To take these possible relationships into account, we included several indicators of social inclusion into the analyses. We controlled for the number of children (continuous in absolute numbers), the number of grandchildren and great-grandchildren (continuous in absolute numbers), the frequency of contact with other people (five-point scale, “never,” “seldom,” “sometimes,” “often,” and “very often”), and the participation in social activities outside the family involving drinking coffee and going out (six-point scale, “never,” “once a year,” “several times a year,” “monthly,” “weekly,” and “daily”).

Other studies (e.g., König et al., 2018) illustrated that individual characteristics influence the use of new technologies in old age. Therefore, we controlled for the age of the individual (continuous in years, mean-centered), gender (ref. female), the level of education (low [ref.], middle, high) according to the classification of education in the German Aging Survey, which was based on the International Standard Classification of Education (ISCED), housing situation (long-term care [ref.], no long-term care), the care level of the individual (no care level [ref.], any care level), and a variable that captures participation in the labor force (never employed [ref.], employed at any point in time).

### Statistical Analyses

SPSS version 25 (IBM Statistics, Amos, NY) was used for the statistical analyses, the missing data were excluded listwise, and the data were weighted using design and poststratification weights. This approach adjusts the age and gender distribution of the sample to the population of NRW and allows extrapolation of the survey results to the state population. Descriptive analyses and regression analyses were applied. We conducted multiple linear regression analyses to explore the relationships between ICT use and loneliness, anomie, and autonomy as dependent variables. In the first step, only the three user groups were included as independent variables. In the second step, the indicators of social inclusion were added as control variables. In the third step, individual characteristics were added as further control variables. This hierarchical structure allowed us to explore whether the effects of ICT use remain stable when controlling for different individual characteristics.

## Results

### Sample Characteristics

In total, 1,698 interviews with individuals aged 80 years and older were analyzed, and we found that 38.5% (*n* = 653) do not use ICT, 35.6% (*n* = 604) use nonweb ICT, and 25.9% (*n* = 440) use web ICT. The mean age was 85.36 years (*SD* = 4.02) with a range from 80 to 103 years. Within the sample, 63.0% (*n* = 1,069) were female, 9.2% (*n* = 156) live in long-term care settings, and 27.6% (*n* = 456) have a care level. According to the ISCED, the majority (53.6%, *n* = 846) had a medium level of education.

The descriptive analyses revealed the differences in ICT use according to individual characteristics (Table 1). For example, men and those who have a higher level of education use web ICT more often than women and individuals with a lower level of education. Furthermore, individuals who were employed at any point in time use web ICT more often than those who were never employed. There are further differences in ICT use based on social inclusion indicators (Table 2). For example, nonusers of ICT have more children than ICT users.

**Table 1.** ICT Use by Individual Characteristics

Total (*n* = 1,698)	% total of the sample	No ICT	Nonweb ICT	Web ICT
		38.5	35.6	25.9
Age (*n* = 1,693)	*M* (*SD*)	86.91 (4.38)	84.73 (3.55)	83.92 (3.26)
Sex (*n* = 1,698)	% female	46.3	35.5	18.2
	% male	25.1	35.8	39.1
Education (*n* = 1,577)	% low level	57.3	34.5	8.2
	% medium level	35.0	38.6	26.4
	% high level	17.8	29.8	52.4
Housing situation (*n* = 1,695)	% private home	34.3	37.7	28.0
	% long-term care	80.1	16.0	3.8
Care level (*n* = 1,651)	% no care level	28.7	39.5	31.7
	% any care level	60.4	26.7	12.9
Participation in the labor force (*n* = 1,695)	% employed at any point in time	60.2	35.9	3.8
	% never employed	37.0	35.6	27.4

*Note*: ICT = information and communication technologies; *M* = mean; *SD* = standard deviation, percentages in rows, proxy interviews excluded.

**Table 2.** ICT Use by Indicators of Social Inclusion

		No ICT	NonWeb ICT	Web ICT
Number of children (*n* = 1,697)	*M (SD)*	2.04 (1.45)	2.14 (1.42)	1.97 (1.27)
Number of grandchildren/great-grandchildren (*n* = 1,681)	*M (SD)*	4.14 (5.82)	3.49 (4.00)	3.13 (3.32)
Frequency: time with other people (*n* = 1,695)	*M (SD)*	2.51 (.95)	2.61 (.96)	2.70 (.85)
Frequency: participation in social activities, e.g., drinking coffee (*n* = 1,695)	*M (SD)*	1.40 (1.75)	1.67 (1.71)	1.76 (1.72)

*Note*: ICT = information and communication technologies; *M* = mean; *SD* = standard deviation, proxy interviews excluded.

### Relation of ICT Use to Loneliness

See Table 3 for means and standard deviations of loneliness in the three groups. ICT use explained a significant amount of variance within the oldest-old’s feeling of loneliness (*F*[2, 1514] = 17.70, *p* < .001, adjusted *R*^2^ = .02). In support of hypothesis 1, nonweb ICT users (*β* = .12, *p* < .001) and nonusers of ICT (*β* = .18, *p* < .001) reported higher levels of loneliness than web ICT users. The effects remain significant when controlled for social inclusion and individual characteristics (nonweb ICT: *β* = .09, *p* = .009; nonusers of ICT: *β* = .08, *p* = .017; Table 4).

**Table 3.** Domains of Subjective Well-Being Within the Different ICT User Groups

	Loneliness (*n* = 1,696)		Anomie (*n* = 1,680)		Autonomy (*n* = 1,691)	
	*M*	*SD*	*M*	*SD*	*M*	*SD*
Total	1.31	0.63	2.48	0.82	3.57	0.71
No ICT	1.41	0.68	2.61	0.85	3.33	0.81
Nonweb ICT	1.30	0.65	2.52	0.83	3.66	0.66
Web ICT	1.18	0.51	2.25	0.73	3.79	0.49

*Note*: ICT = information and communication technologies; *M* = mean; *SD* = standard deviation, proxy interviews excluded.

**Table 4.** Linear Regression Analyses to Predict Loneliness

	*b (SE)*	*Β*	*p*
**Model 1: ICT only**			
No ICT (ref. Web ICT)	.24 (.04)	.18	<.001
Nonweb ICT (ref. Web ICT)	.16 (.04)	.12	<.001
Model fit	*F*(2, 1514) = 17.70, *p* < .001, adjusted *R*^2^: .02		
**Model 2: indicators of social inclusion as covariates**			
No ICT (ref. Web ICT)	.22 (.04)	.16	<.001
Nonweb ICT (ref. Web ICT)	.14 (.04)	.11	<.001
Model fit	*F*(6, 1510) = 20.24, *p* < .001, adjusted *R*^2^: .07		
**Model 3: indicators of social inclusion and individual characteristics as covariates**			
No ICT (ref. Web ICT)	.11 (.05)	.08	.017
Nonweb ICT (ref. Web ICT)	.11 (.04)	.09	.009
Model fit	*F* (13, 1503) = 13.00, *p* < .001, adjusted *R*^2^: .09		

*Note*: *b* = unstandardized regression coefficients; ICT = information and communication technologies; *SE* = standard errors; *β* = standardized regression coefficients; model 2 includes, as covariates the number of children, number of grandchildren/great-grandchildren, frequency of time spent with other people, and frequency of social activities; model 3 include, as additional covariates, age (mean-centered), sex (ref. female), education level (ref. low level), care level (ref. no care level), long-term care (ref. private home), and participation in the labor force (ref. never employed).

### Relation of ICT Use to Anomie

See Table 3 for means and standard deviations of anomie in the three groups. ICT use explained a significant amount of variance within the oldest-old’s feeling of anomie (*F*[2, 1509] = 22.43, *p* < .001, adjusted *R*^2^ = .03). In support of hypothesis 2, users of nonweb ICT (*β* = .16, *p* < .001) and nonusers of ICT (*β* = .20, *p* < .001) reported higher levels of anomie than users of web ICT. The effects remained significant when controlled for social inclusion and individual characteristics (nonweb ICT: *β* = .12, *p* < .001; nonusers of ICT: *β* = .10, *p* = .004; Table 5).

**Table 5.** Linear Regression Analyses to Predict Anomie

	*b (SE)*	*Β*	*p*
**Model 1: ICT only**			
No ICT (ref. Web ICT)	.34 (.05)	.20	<.001
Nonweb ICT (ref. Web ICT)	.27 (.05)	.16	<.001
Model fit	*F*(2, 1509) = 22.43, *p* < .001, adjusted *R*^2^: .03		
**Model 2: indicators of social inclusion as covariates**			
No ICT (ref. Web ICT)	.30 (.05)	.17	<.001
Nonweb ICT (ref. Web ICT)	.25 (.05)	.15	<.001
Model fit	*F*(6, 1505) = 15.54, *p* < .001, adjusted *R*^2^: .06		
**Model 3: indicators of social inclusion and individual characteristics as covariates**			
No ICT (ref. Web ICT)	.17 (.06)	.10	.004
Nonweb ICT (ref. Web ICT)	.21 (.05)	.12	<.001
Model fit	*F*(13, 1498) = 9.68, *p* < .001, adjusted *R*^2^: .07		

*Note*: *b* = unstandardized regression coefficients; ICT = information and communication technologies; *SE* = standard errors; *β* = standardized regression coefficients; model 2 includes, as covariates the number of children, number of grandchildren/great-grandchildren, frequency of time spent with other people, and frequency of social activities; model 3 include, as additional covariates, age (mean-centered), sex (ref. female), education level (ref. low level), care level (ref. no care level), long-term care (ref. private home), and participation in the labor force (ref. never employed)

### Relation of ICT Use to Autonomy

See Table 3 for means and standard deviations of autonomy in the three groups. ICT use explained a significant amount of variance within the oldest-old’s feeling of autonomy (*F*[2, 1514] = 54.38, *p* < .001, adjusted *R*^2^ = .07). In support of hypothesis 3, users of nonweb ICT (*β* = −.12, *p* < .001) and nonusers of ICT (*β* = −.31, *p* < .001) reported lower levels of autonomy than users of web ICT. The effects remained significant when controlled for social inclusion and individual characteristics (nonweb ICT: *β* = −.09, *p* = .003; nonusers of ICT: *β* = −.16, *p* < .001; Table 6).

**Table 6.** Linear Regression Analyses to Predict Autonomy

	*b (SE)*	*Β*	*p*
**Model 1: ICT only**			
No ICT (ref. Web ICT)	−.45 (.04)	−.31	<.001
Nonweb ICT (ref. Web ICT)	−.18 (.05)	−.12	<.001
Model fit	*F*(2, 1514) = 54.38, *p* < .001, adjusted *R*^2^: .07		
**Model 2: indicators of social inclusion as covariates**			
No ICT (ref. Web ICT)	−.43 (.04)	−.29	<.001
Nonweb ICT (ref. Web ICT)	−.17 (.04)	−.12	<.001
Model fit	*F* (6, 1510) = 26.13, *p* < .001, adjusted *R*^2^: .09		
**Model 3: indicators of social inclusion and individual characteristics as covariates**			
No ICT (ref. Web ICT)	−.23 (.05)	−.16	<.001
Nonweb ICT (ref. Web ICT)	−.13 (.04)	−.09	.003
Model fit	*F*(13, 1503) = 35.28, *p* < .001, adjusted *R*^2^: .23		

*Note*: *b* = unstandardized regression coefficients; ICT = information and communication technologies; *SE* = standard errors; *β* = standardized regression coefficients; model 2 includes, as covariates the number of children, number of grandchildren/great-grandchildren, frequency of time spent with other people, and frequency of social activities; model 3 include, as additional covariates, age (mean-centered), sex (ref. female), education level (ref. low level), care level (ref. no care level), long-term care (ref. private home), and participation in the labor force (ref. never employed).

## Discussion

This research is among the first to analyze the relationship between ICT use and subjective well-being in the oldest-old. A distinctive element of our study was that the findings are based on a representative sample of individuals aged 80 years and older in private homes and institutional settings. We found significant relationships between the use of web-connected ICT and the three domains of subjective well-being: using web-connected ICT was significantly related to loneliness, anomie, and autonomy. Participants who use web-connected ICT reported lower levels of loneliness, lower levels of anomie, and higher levels of autonomy. These results are consistent with our hypotheses and the results reported in previous research (e.g., Allaire et al., 2013; Castellacci & Tveito, 2018; Cotten et al., 2013; Sims et al., 2017). Our findings also fit the conclusion that new technology is more likely to expand traditional forms of social interaction than to replace them (Antonucci, et al., 2017). Among the three domains of subjective well-being, the prediction of autonomy showed the highest levels of explained variance. The low levels of *R*^2^ are comparable to previous studies on ICT and well-being in the oldest-old (Sims et al., 2017). Despite low levels of explained variance, the use of web-connected ICT contributed to the three domains of well-being even when controlling for indicators of social inclusion and individual characteristics. In the final models, 7%–23% of the variance in well-being was explained by ICT use and the control variables. Our results indicate that the use of ICT can have an independent effect on selected domains of well-being in old age, and a significant part of loneliness, anomie, and autonomy is explained by the use of ICT devices. In this way, our findings provide new insights into the relevant role of ICT use in very old age and illustrate positive relationships between today’s modern ICT devices and subjective experiences of well-being.

Our study results have demonstrated that the oldest-old generally use ICT in their everyday life. More than half the participants used at least one ICT device, and about every fourth person used web-connected ICT. However, the web-connected ICT group was the smallest group within the whole sample, which fits the conclusion that an age-related digital divide still exists (Mitzner et al., 2019). The usage rates of web-connected ICT were lower in our study when compared with those of the general population (e.g., IT.NRW, 2019; Pew Research Center, 2018) as well as among younger-old cohorts (e.g., König et al., 2018). Our descriptive analyses indicate differences in ICT use according to individual characteristics: individuals who live in long-term care, with lower levels of education, who are female, and who are older use web ICT less often than individuals who live in private homes, who are better educated, younger, and male. This finding is consistent with previous research among younger-old cohorts (e.g., König et al., 2018) but should be studied in more detail in future work.

Our results provide further evidence that a good person–environment-fit can contribute to subjective well-being in old age and that technology is a relevant context characteristic in old age. We present a novel finding that the use of web-connected ICT is a relevant person–technology interaction among the oldest-old and its use is positively associated with different domains of subjective well-being.

These findings need to be discussed on several levels. There might be selection effects in the composition of the three groups. Learning to use new technologies is associated with greater effort, and the oldest old often have to overcome barriers arising from fewer cognitive, physical, financial, and social resources (e.g., Czaja et al., 2006; Schulz et al., 2015). We controlled for some individual characteristics and some indicators of social inclusion. There might be further effects that we could not control for. Individuals with better functionality are likelier to use web-connected ICT. Elliot et al. (2014) and Forsman et al. (2017) demonstrated that ICT use is related to mental health and well-being in old age. These studies indicated that there are complex relationships and several link mechanisms between ICT use and well-being.

To stress the general ICT use patterns and relationships, we considered a global measurement of nonweb-connected ICT use and web-connected ICT use. Depending on the intended use, the effects on subjective well-being may be different. For example, future studies could also consider whether there are specific effects of single devices compared with the more general effect of web-connected ICT. It is important to study these relationships in future work to gain more information on how ICT use impacts subjective well-being in more detail, using systematic approaches. One specific aspect of interest might be to investigate online social networking sites. Hajek and König (2019) demonstrated that daily users of online social network sites over the age of 40 reported lower levels of social isolation. At the same time, negative effects such as superficial relationships and stress are reported in other studies (e.g., Castellacci & Tveito, 2018).

In addition, the duration of use and past experiences with ICT devices should be considered. Understanding the successful use of ICT over time is important because sustained use provides the opportunity to profit from the benefits of technology in a digital society (Cotten, 2017). As far as we could ascertain, there are no studies that have analyzed these relationships in oldest-old individuals. Older adults often use technology selectively and in unexpected ways, and develop their own digital skills and strategies (Peine & Neven, 2019). Research on aging and technology should consider this usage behavior as legitimate and not as mistakes or wrong usage because it helps to understand the role of technology in older adults’ everyday lives (Peine & Neven, 2019).

Studying person–environment interchange processes has a long and well-established research tradition in gerontological research (Wahl et al., 2012) but the field of environmental aging and technology research is still an emerging research field (Wahl & Gerstorf, 2018). Since the theoretical discussion of aging and technology is still in its infancy (Schulz et al., 2015), the results of the present study should be tested and refined in future works. Future research should also consider the relevance of contextual factors for technology adoption in more detail. Context information should be included to enhance ecological validity of survey data (Wilkinson, Ferraro, & Kemp, 2017). On a methodological level, this may include information from surveys in everyday life, for example, by using ambulatory assessments and digital diary studies. According to Wahl and Gerstorf (2018), “ambulatory assessments provide entirely new avenues for naturalistic research to empirically test the noted theoretical predictions about how people select into, actively shape, and make use of the contexts of their everyday lives” (p. 172).

## Implications

In addition to the observed positive relationship between subjective well-being and the use of technology, the possible negative effects of ICT use must not be ignored. Using technology might lead to stress, and the older users may feel overburdened when handling technology (Wahl & Gerstorf, 2018). To support older adults who wish to use web-connected ICT and to avoid negative effects, it would be helpful to offer support and training to increase their self-efficacy and digital literacy skills. The special learning needs of older adults must also be considered in the design of technology (Czaja, Boot, Charness, & Rogers, 2019), with attention paid to their technological skills and their physical limitations, such as impaired vision or difficulties with fine motor skills.

However, there are multiple reasons why older adults engage or do not engage in technologies, and they actively choose their preferred technology (Peine & Neven, 2019). The mere objective fact that a person does not have access to a specific technology like the Internet does not imply a subjective feeling of exclusion (Seifert et al., 2018). An unintentional termination of Internet use is more likely when an older adult moves from a private home to a nursing home (Seifert, Doh, & Wahl, 2017). In addition to offering training, digital infrastructure should be available in nursing homes to avoid the negative effects of involuntary nonuse of ICT.

## Limitations

There were some limitations to the study design and analytical approach. For some concepts (i.e., loneliness and autonomy), single-item measurements were applied that should be evaluated in future studies. The representative survey was conducted in NRW, Germany only. The state might differ from other regions around the world in terms of population density and the diffusion of Internet technology. The study was a cross-sectional survey, and we can only identify patterns and correlations and no causal relationships. For example, higher levels of autonomy may facilitate the use of web-connected ICT. Longitudinal approaches could be used to analyze the effects of cohort flow in the adoption of new technologies. These longitudinal approaches could also be used to ascertain the test–retest reliability for the single-item measurements of loneliness and autonomy.

No analysis on intensity of use was possible with the existing dataset. Furthermore, purposes of and prior experiences with ICT use, and digital skills were not measured; hence, we could not analyze the level of digital expertise and literacy. It is likely that the subjective well-being domains are differently related to specific aspects of ICT use, for example, whether ICT use is more instrumental or for entertainment. In order to validate our findings, they need to be replicated and compared with other studies on technology adoption among the oldest-old in the future.

## Conclusion

This study reports the results on ICT use of the first representative state-wide survey study of the oldest-old in NRW, Germany. The findings shed light on the relationships between ICT use and subjective well-being in an underexplored group. With our study, we were able to demonstrate that the use of web-connected ICT is related to the subjective well-being domains of loneliness, anomie, and autonomy. As more research examines the implications of ICT use, it is important to pay attention to its effect on the well-being of the oldest-old.

## Compliance with Ethical Guidelines

All processes of the study were in accordance with the ethical standards of the ethics committee of the Medical Faculty of the University of Cologne (No. 17-169) and with the Helsinki Declaration of 1975 (in its most recently amended version). Informed consent was obtained from all of the participants included in the study.

## Conflicts of Interest

None reported.
